# Spinal dural ossification causing neurological signs in a cat

**DOI:** 10.1186/1751-0147-55-47

**Published:** 2013-06-19

**Authors:** Johanna M Antila, Janis Jeserevics, Mindaugas Rakauskas, Marjukka Anttila, Sigitas Cizinauskas

**Affiliations:** 1 The Small Animal Hospital Aisti, Virtatie 9, Vantaa, FI-01600, Finland; 2 Pathology Unit, Finnish Food Safety Authority Evira, Mustialankatu 3, Helsinki FI-00790, Finland

**Keywords:** Neurology-small animal, Cat, Dural ossification, Osseous metaplasia, Tetraparesis

## Abstract

A six-year-old Ragdoll cat underwent examination due to a six-month history of slowly progressive gait abnormalities. The cat presented with an ambulatory tetraparesis with a neurological examination indicating a C1-T2 myelopathy. Radiographs of the spine showed a radiopaque irregular line ventrally in the vertebral canal dorsal to vertebral bodies C3-C5. In this area, magnetic resonance imaging revealed an intradural extramedullary/extradural lesion compressing the spinal cord. The spinal cord was surgically decompressed. The cause of the spinal cord compression was dural ossification, a diagnosis confirmed by histopathological examination of the surgically dissected sample of dura mater. The cat gradually improved after the procedure and was ambulating better than prior to the surgery. The cat’s locomotion later worsened again due to ossified plaques in the dura causing spinal cord compression on the same cervical area as before. Oral prednisolone treatment provided temporary remission. Ten months after surgery, the cat was euthanized due to severe worsening of gait abnormalities, non-ambulatory tetraparesis. Necropsy confirmed spinal cord compression and secondary degenerative changes in the spinal cord on cervical and lumbar areas caused by dural ossification. To our knowledge, this is the first report of spinal dural ossification in a cat. The reported cat showed neurological signs associated with these dural changes. Dural ossification should be considered in the differential diagnosis of compressive spinal cord disorders in cats.

## Background

Spinal dural ossification (i.e. osseous dural metaplasia, ossifying pachymeningitis) is a relatively common finding in dogs, particularly in middle-aged or older dogs [1]. It is usually an incidental finding, but in rare cases has presumably caused clinical signs such as hyperesthesia of one or both thoracic limbs, pain, weakness, progressive paresis, ataxia, atrophy of the limb musculature, urinary retention, and incontinence [1-8]. In humans, dural ossification is frequently observed at autopsy and surgery [9]. These dural findings are usually clinically unremarkable, but in rare cases have been associated with neurological deficits [9,10]. In humans, dural ossification has been associated with chronic arachnoiditis, spinal cord injury, ossification of the ligamentum flavum, and ossification of the posterior longitudinal ligament [10-13]. The terms osseous dural metaplasia and ossifying pachymeningitis refers to the assumed etiology of this condition. However, the etiology of dural ossification remains unknown [1,4]. Spinal dural ossification has not previously been reported in other animals than dogs. This is the first report of spinal dural ossification in a cat. This is also the first report of spinal dural ossification associated with neurological signs in a cat. This paper describes the diagnostic work-up, surgical treatment and outcome in a cat with neurological signs due to spinal dural ossification.

## Case presentation

### History

A six-year-old 6-kg castrated male Ragdoll cat was admitted to the Small Animal Hospital Aisti, Finland, after a six-month history of slowly progressive tetraparesis. Over the six-month period, the cat developed gait abnormalities, beginning with slight weakness of the pelvic limbs to ambulatory tetraparesis with moderate generalized ataxia.

The cat had taken a fall from a height of approximately 1.5 meters during the time the owner had noticed the first neurological signs. The cat had remained indoors and received homemade food with appropriate amounts of nutrients, vitamins and minerals [14,15]. The cat was routinely vaccinated and dewormed according to national recommendations. One week prior to admission, blood values (hematology and biochemistry) were checked. All values were within the reference range.

### Clinical findings

The results of general physical and orthopedic examinations were unremarkable. Neurological examination revealed ambulatory tetraparesis and moderate generalized ataxia. Postural reactions were decreased in all four limbs, more on the left side. Except for a mildly decreased flexor reflex in the thoracic limbs, spinal reflexes were otherwise normal. Cranial nerves were normal, the cat showed no signs of pain during palpation of the back or manipulation of the neck. The neurological examination indicated that neuroanatomical localization was at the C1-T2 spinal cord segments. Differential diagnoses included neoplastic, inflammatory, infectious and degenerative diseases. A urinary sample and a new blood sample were taken. The results of the urinalysis, hematology and serum biochemistry were unremarkable.

### Diagnostic imaging

**Figure 1:**
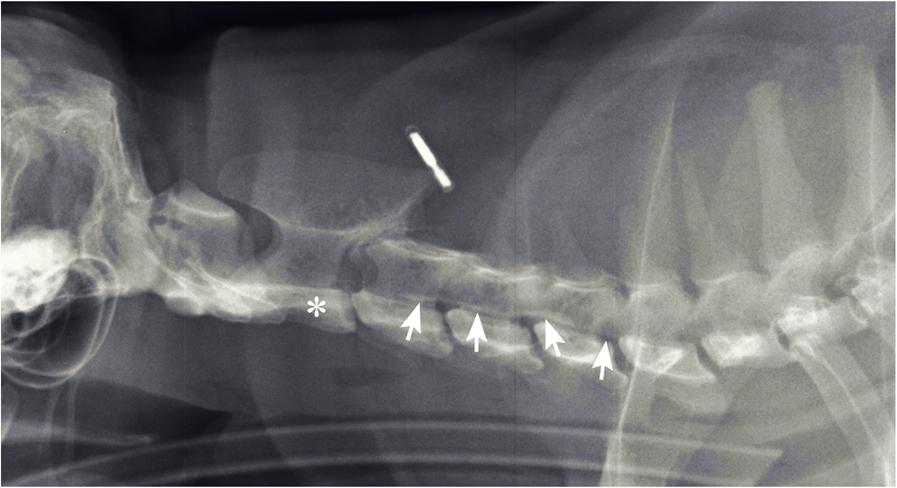
**Lateral radiograph of the cervical vertebral column.** Thin radiopaque line (pointer arrows) appears ventrally in the vertebral canal dorsal to vertebral bodies C3-5. The asterisk corresponds to the second cervical vertebra.

Lateral radiographs of the spine revealed a radiopaque irregular line ventrally in the vertebral canal dorsal to vertebral bodies C3-C5 (Figure 1).

**Figure 2:**
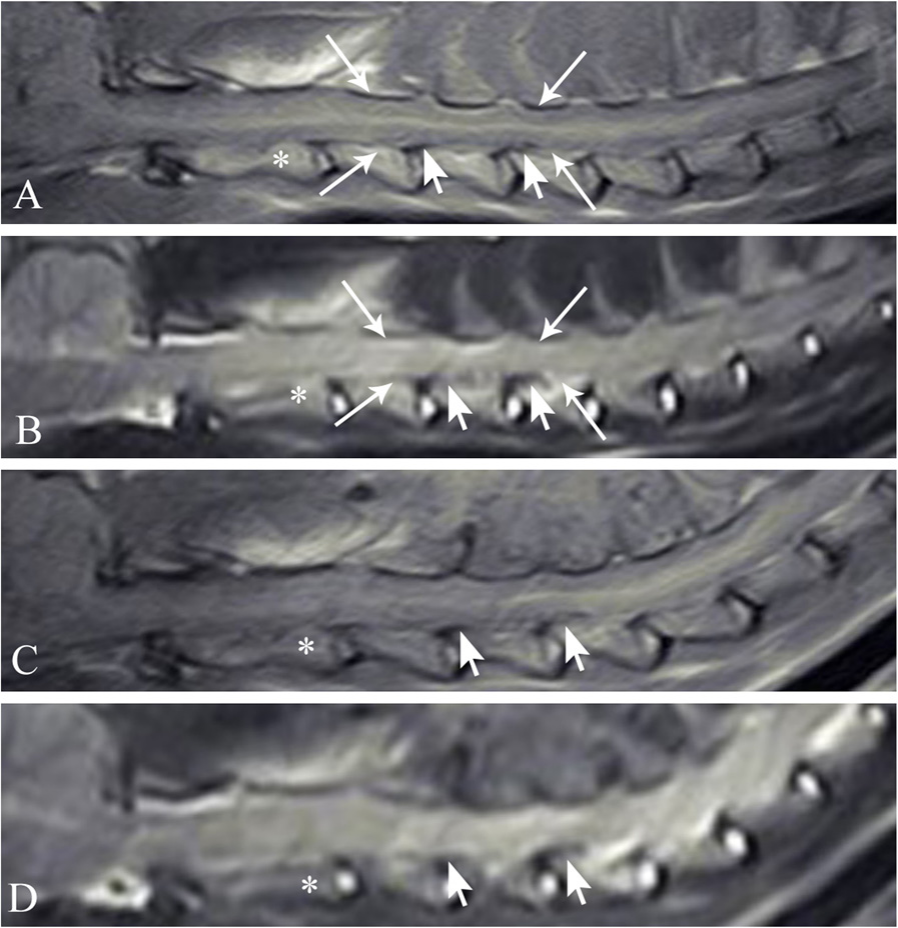
**Sagittal planes of magnetic resonance images T1-weighted (A), T2-weighted (B) before surgery and T1-weighted (C), T2-weighted (D) after surgery.** Intradural extramedullary/extradural hypointensity in the vertebral canal (pointer arrows) in T1-weighted and T2-weighted images corresponds to a radiopaque line evident in the lateral radiograph at the level of C3-5. The arrows depict stenosis of the vertebral canal and flattening/compression of the spinal cord; the asterisk denotes the C2 vertebra.

**Figure 3:**
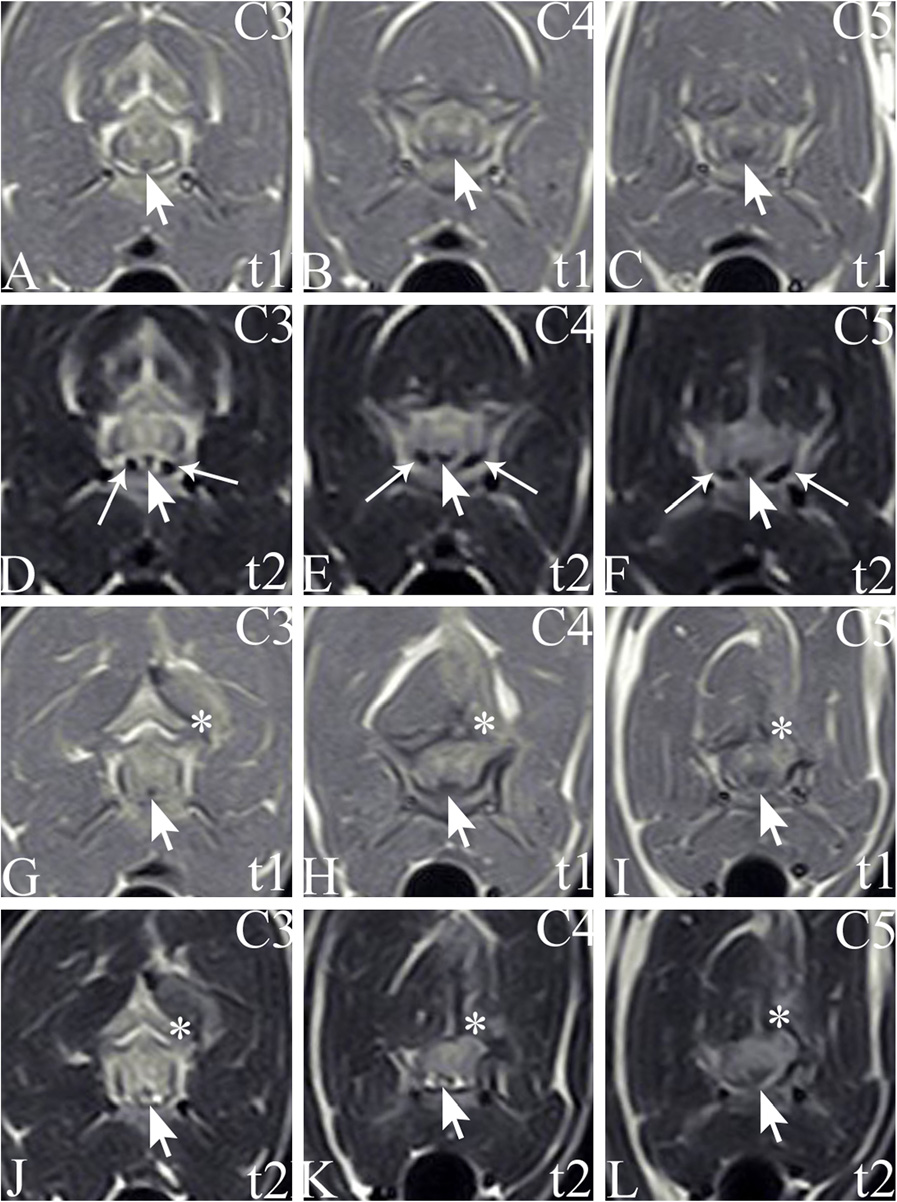
**Transversal planes of magnetic resonance images before surgery (A, B, C, D, E, F) and after surgery (G, H, I, J, K, L).** The symbols in the right upper corner encode images taken at the level of the third cervical vertebra (C3), the fourth cervical vertebra (C4) and the fifth cervical vertebra (C5). Intradural extramedullary/extradural hypointensity (pointer arrows) in T1W (t1) and T2W (t2) is causing ventrodorsal spinal cord flattening and compression. The asterisk shows post operative scar tissue, the arrows show the ventral sinuses.

Magnetic resonance imaging (MRI) of the neck was performed.^a^ Images were obtained in sagittal and transverse planes. Sequences included T1-weighted (T1W) and T2-weighted (T2W). In the sagittal and transverse T1W and T2W images, intradural extramedullary/extradural hypointensity was causing ventrodorsal spinal cord flattening and compression at the level of vertebral bodies C3-C5 (Figures 2A-B, 3A-F). Contrast enhancement was not visible after an IV bolus of contrast medium (gadopentetate dimeglumine 0.1 mmol/kg, IV). The suspected diagnosis was spinal dural ossification. Due to spinal cord compression, the treatment selected was surgical.

### Surgical treatment

The cat underwent a hemilaminectomy from the left side from C3 to C5. The dura mater was sharply dissected from the left, dorsal and ventral part of the underlying spinal cord at the level of vertebral bodies C3-C5. The dura mater was thicker, harder, paler and more opaque in the diseased area than in the healthy area, which was at the border of the dissection. The spinal cord appeared to be mildly atrophied, but was otherwise normal.

### Histopathological examination of the biopsy

Samples of the dura mater were collected from the lesion site during surgical revision, fixed in 10% buffered formalin and sent for histopathological evaluation (Veterinary pathology lab. PatoVet, Helsinki, Finland).

The samples of the dura mater were routinely processed, embedded in paraffin, sectioned at 4 μm and stained with haematoxylin and eosin. The sections consisted of collagen-rich mature fibrous connective tissue which contained islands of calcified bone and cartilage multifocally as well as occasional lymphocytes. The findings were typical of dural ossification.

### Postoperative development

After surgery, the cat received antibiotic amoxicillin clavulanic acid (17 mg/kg, PO, q 12 h for 10 days), a fentanyl transdermal patch (25 ug/h for 3 days) and non-steroidal anti-inflammatory drug meloxicam (0.05 mg/kg, PO, q 24 h as needed). The cat also underwent physiotherapy. The cat returned home two days after surgery. With non-ambulatory tetraparesis, however, its neurological status was worse than before the surgery.

In the first weeks after surgery, the neurological status of the cat improved considerably to a point such that the gait was better than prior to surgery. Forty days after surgery, however, the cat deteriorated and was returned to the hospital. Its neurological status was better than prior to the surgery, but not normal. The cat presented with generalized ataxia with no obvious paresis. Postural reactions were decreased on the left side. The cat showed no signs of pain. A lesion was localized to the C1-T2 spinal cord segments.

Another MRI of the cervical spine was performed. Again, in the transverse and sagittal T1W and T2W images, intradural extramedullary/extradural hypointensity was causing ventrodorsal spinal cord flattening and compression at the level of vertebral bodies C3-C5. Additionally, in the T1W and T2W sequences, a roundish structure along the entire length of the previous hemilaminectomy site was identified as isointense. The structure caused no significant spinal cord compression, however, and was interpreted as scar tissue (Figures 2C-D, 3G-L).

No second surgery was recommended due to the risk for spinal cord damage, as it had no dura mater covering in this area. Additionally, adhesions between the scar tissue in the hemilaminectomy wound and the pia mater were likely. Therefore, treatment with corticosteroids (prednisolone 1 mg/kg, PO, q 24 h for one week, then 0.5 mg/kg, PO, q 24 h for one week, then 0.25 mg/kg, PO, q 24 h) was initiated instead.

The cat improved with prednisolone treatment and was ambulating better, but still not completely normally. Six and a half months after surgery, the cat’s locomotion worsened. The prednisolone dosage was increased to 1 mg/kg, PO, q 24 h. The cat’s neurological status improved for one week, but then progressively worsened. The prednisolone dosage was then increased to 1.5 mg/kg, PO, q 24 h. Ten months after surgery, the cat was euthanized due to severe worsening of gait abnormalities, non-ambulatory tetraparesis, and was sent for necropsy (Pathology Unit, Evira, Helsinki, Finland).

### Necropsy findings

**Figure 4:**
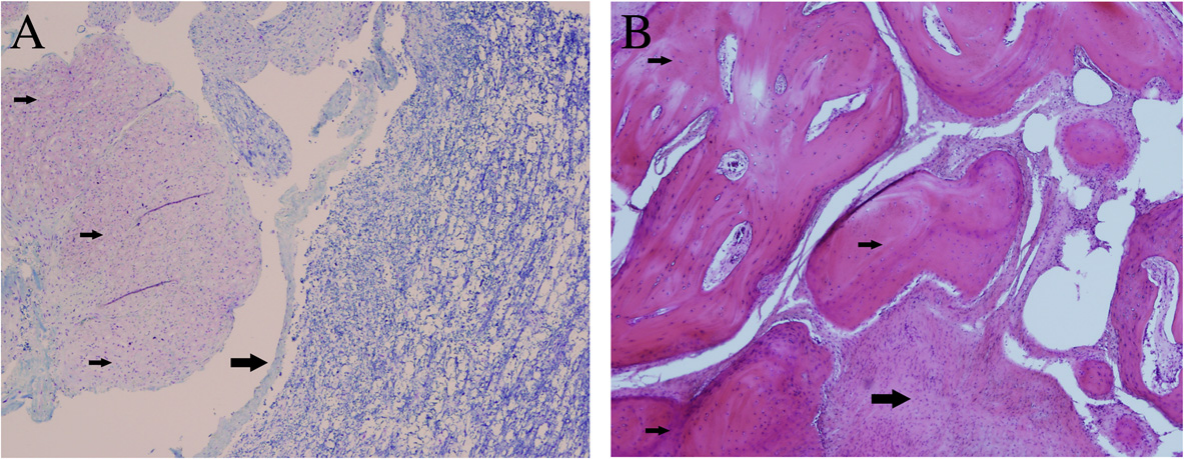
**Longitudinal section of the cervical spinal cord (A).** The fibrous connective tissue with chondroid metaplasia (small arrows) markedly thickens the dura. The spinal cord shows depression in the corresponding area (large arrow). Luxol fast blue, ×4. Longitudinal section of the dura mater of the cervical spine (**B**). The plaques are composed of collagen-rich connective tissue (large arrow) and well-organized bone trabeculae (small arrows). Haematoxylin and eosin, ×4.

A complete necropsy was performed. Samples of all major organs and the brain and spinal cord were fixed in 10% buffered formalin. The fixed samples were routinely processed, embedded in paraffin, sectioned at 4 μm, and stained with haematoxylin and eosin, luxol fast blue and the Bielschowsky method. Specific lesions were limited to the central nervous system. Marked multifocal to coalescing dural ossification was evident in the C3-C5 and caudal lumbar areas of the dura mater. The ossified and occasionally chondroid plaques were located ventrally to the spinal cord and were as much as 3 mm thick. The plaque surfaces facing the spinal cord were uneven and rough, and there were multifocal depressions in the ventral funiculi of the spinal cord (Figure 4A). The thick plaques consisted of well-differentiated trabecular bone with marrow constituents and collagen-rich connective tissue (Figure 4B). Multifocally, there were small islands of cartilage and mild lymphocytic infiltration. The affected areas of the spinal cord showed multifocal atrophy of the ventral funiculi corresponding to the depressions observed macroscopically. Some areas showed the marked edema of individual myelin sheaths with occasional axonal spheroids and macrophages. Surrounding the C3-C5 and caudal lumbar areas of the dura mater were milder changes consisting of multifocal chondroid metaplasia and calcification of the dura mater. No specific macroscopic lesions were observed at the hemilaminectomy site. An incidental finding was a syncytial meningioma, 1 mm in diameter, in the caudal commissure of the cerebrum.

## Discussion

To the best of our knowledge, this is the first report of spinal dural ossification in a cat. Cats may have dural ossification at higher frequencies than expected because routine radiographs can sometimes overlook small ossified plaques. In one study, radiographs of dogs overlooked plaques smaller than 2 mm in diameter [6]. In radiographs, ossified plaques can be confused with calcified herniated intervertebral disk material, lateral spondylosis deformans, the accessory processes of vertebral bodies or vertebral osteophytes [6,16]. Clarifying the incidence of dural ossification in cats will require more investigation. Spinal dural ossification seldom causes clinical disease in dogs, and the clinical value in cats may be roughly the same.

When visible, dural ossification is evident in native radiographs as a radiopaque line along the vertebral canal, most easily detected in areas of the intervertebral foramina and ventrally to the spinal cord [3,16]. The radiographs of the cat showed a clearly visible radiopaque line in the affected cervical area. When dural ossification occured in the lumbar area remains unclear as no radiographic changes were observed in that area. Myelographic examination or advanced imaging techniques, such as MRI or computed tomography, have proved useful in defining the clinical importance of the radiographic findings and help in selecting the appropriate treatment protocol [1,8]. In the reported cat, the rather low resolution of the MR images could not show whether the hypointense lesion, which was compressing the spinal cord, was intradural extramedullary or extradural. The suspected diagnosis was spinal dural ossification, and the differential diagnosis was neoplasia (e.g. a nerve sheath tumor, lymphoma, meningioma). Taking in to account several conditions, such as low-field MRI^a^, low image resolution, relatively small patient size, interpreting images is challenging. The lesion was hypointense in the T1W and T2W sequences. Usually such a pattern suggests mineralization of the tissue, more collagen fibers or old bleeding. Non-specific mineralization or ossification may sometimes occur in tumors. Based only on the absence of contrast enhancement, neoplasia could not be ruled out. Surgery and later necropsy confirmed an intradural extramedullary lesion: dural ossification. Surgery is justified if spinal cord compression is associated with dural ossification [1].

Surgical decompression of the spinal cord was perormed in the cat, but the procedure offered only temporary remission. Consequently, clinical signs may recur in the future, especially if some parts of the compressive ossified plaques remain after surgery, and these remnants continue to grow. Plaque formation may recur after surgery. Additionally, dural ossification may occur and cause spinal cord compression in other areas. The second MRI of the cat revealed spinal cord compression in the same cervical area as before the surgery, most likely due to remnants of unsuccessfully removed compressive ossified plaques. Presumably, these remnants grew in the time after surgery, thus causing deterioration of the cat. Later, the necropsy of the cat confirmed spinal cord compression due to ossified plaques in the same cervical area as before the surgery as well as in the lumbar dura mater. These necropsy findings explain the cat’s deterioration. Excessive scar tissue formation in the area of surgery may occur, which in itself may cause neurological signs by compressing the spinal cord. In the necropsy of the cat, excessive scar tissue that was compressing the spinal cord was not identified. Prednisolone treatment may improve clinical signs, as occurred in the cat, possibly by reducing inflammatory reaction and edema in the compressed spinal cord [17]. Later, despite the prednisolon treatment, the neurological status of the cat deteriorated, likely because the slowly increasing compression of the spinal cord caused degeneration of the nerve fibers and myelin sheaths. This compression resulted in a narrowing of the spinal cord due to the loss of fibers and their myelin sheaths. Immediately after surgery, the temporarily deteriorated neurological status of the cat may have occured due to reperfusion injury [18] or iatrogenic injury to the spinal cord [19].

Some researchers suggest that dural ossification may be a form of direct metaplasia of the connective tissue of the dura mater. Consequently, dural ossification is also known as “osseous dural metaplasia”. Dural ossification is characterized by the deposition of ossified plaques (i.e. lamellar bone plaques) in the inner surface of the dura [7,20]. These grayish islands contain red marrow surrounded by cortical bone [3,6]. Heterotopic osteoblasts, suspected to be important in this metaplastic process [21,22], originate from connective tissue cells derived most probably from undifferentiated mesenchymal cells with osteogenic potential, termed inducible osteogenic precursor cells [21,22]. In the cat, histopathological evaluation of the dura mater also revealed cartilage formation. The ossified plaques may thus have resulted from osseous metaplasia or endochondral ossification of the cartilage.

Ossified plaques are usually located along the midline of the dura, ventral to the spinal cord [23]. Many studies of dogs find that ossification occurs mainly in the cervical and lumbar areas of the dural tube [6,7]. Additionally, in the cat, changes occured in the cervical and lumbar regions, along the midline of the dura, ventral to the spinal cord.

In general, the etiology of dural ossification remains obscure [1,4]. Cervical and lumbar regions, where changes usually occur, are the most movable regions of the spinal column. However, whether mechanical causes influence the process remains unclear [7]. Focal dural ossified plaques may occur if the dura mater rubs against adjacent vertebral structures (e.g. articular facets) or secondary to other spinal cord disorders, such as disk protrusion [24]. Although the lesion has been called “ossifying pachymeningitis”, thus far no studies have shown that ossification results from inflammation [5,23]. In humans, it has been suggested that arachnoiditis ossificans represents end-stage chronic arachnoiditis [11]. The inflammation may, however, be part of the process that causes ossification. Both the surgically dissected and necropsy samples of the cat showed only mild chronic inflammatory reaction. At the time of sampling, only chronic changes in the spinal cord could account for the clinical signs, which resulted from chronic compression by the plaques. More acute lesions would have occurred previously, though at the time of clinical presentation, they did not account for the clinical signs. Nor could spinal dural ossification be attributed to any other compressive or instability disorder of the spinal cord. The cat had fallen from a stand approximately 1.5 meters high at about the same time the first signs of gait abnormalities appeared. The owner could not say whether the signs began before or after the accident. Trauma as an initiating factor of dural ossification is unlikely, but not impossible in this case. In dogs, spinal dural ossification is considered an age-related degenerative process [4,25]. Age is unlikely to have significantly influenced the development of dural ossified plaques because the cat was only six years old when the clinical signs appeared. The necropsy of the cat revealed a minor meningioma in the cerebrum, a finding associated with no neurological signs and believed to have no association with the dural ossification in the cat.

## Conclusions

In conclusion, although rare in cats, dural ossification can occur and can be associated with neurological signs. Dural ossification should therefore be considered in the differential diagnosis of compressive spinal cord disorders in cats. Surgery is recommended in focal spinal cord compression, but may offer only temporary remission.

## Consent

Written informed consent was obtained from the owner of the animal.

## Endnotes

^a^Esaote Vet MR system with a magnetic field strength of 0.2 Tesla and a DPA coil, Esaote S.p.A., Genoa, Italy.

## Abbreviations

MRI: Magnetic resonance imaging; PO: Per os.

## Competing interests

The authors declare that they have no competing interests.

## Authors’ contributions

JMA carried out the clinical examination and is the main author of this paper. JJ and MR carried out the diagnostic imaging. JJ performed the surgery. MA is responsible for the histopathological and pathological examination parts of the manuscript. SC reviewed the draft. All authors have read and approved the final manuscript.
